# Depressive and anxiety symptoms in the course of the COVID-19 pandemic among physicians in hospitals: results of the longitudinal, multicenter VOICE-EgePan survey over two years

**DOI:** 10.1186/s40359-023-01354-5

**Published:** 2023-10-10

**Authors:** Eva Morawa, Werner Adler, Caterina Schug, Franziska Geiser, Petra Beschoner, Lucia Jerg-Bretzke, Christian Albus, Kerstin Weidner, Andreas M. Baranowski, Yesim Erim

**Affiliations:** 1 Department of Psychosomatic Medicine and Psychotherapy, University Hospital of Erlangen, Friedrich-Alexander University Erlangen-Nürnberg (FAU), Schwabachanlage 6, Erlangen, 91054 Germany; 2 Institute of Medical Informatics, Biometry, and Epidemiology, Friedrich-Alexander University Erlangen-Nürnberg (FAU), Erlangen, Germany; 3 Department of Psychosomatic Medicine and Psychotherapy, University Clinic of Bonn, Bonn, Germany; 4 Department of Psychosomatic Medicine and Psychotherapy, Ulm University Medical Center, Ulm, Germany; 5 Department of Psychosomatics and Psychotherapy, Medical Faculty, University Hospital of Cologne, Cologne, Germany; 6 Department of Psychotherapy and Psychosomatic Medicine, Faculty of Medicine, Technische Universität Dresden, Dresden, Germany

**Keywords:** Anxiety, COVID-19, Depression, Health care workers, Hospital, Mental health, Physicians

## Abstract

**Background:**

This longitudinal, multicenter web-based study explored the trajectories of depressive and anxiety symptoms during the COVID-19 pandemic among physicians over two years.

**Methods:**

At four measurement points between 4/2020 and 5/2022 depressive (Patient Health Questionnaire-2, PHQ-2) and anxiety symptoms (Generalized Anxiety Disorder Scale-2, GAD-2) among physicians in German hospitals were assessed. Time, gender and age effects were analyzed with linear mixed regression models. Comparisons with norm values for the German population during the COVID-19 pandemic were also performed and frequencies of probable depression and anxiety are reported.

**Results:**

The physicians (N = 340) showed a significant increase of depressive symptoms from T1 (M = 1.35, SD = 1.33) to T4 (M = 1.64, SD = 1.34) (p < .001) and of anxiety symptoms from T1 (M = 1.35, SD = 1.42) to T2 (M = 1.59, SD = 1.43) (p = .024). The main effect of gender was only significant for anxiety symptoms (p = .001): women demonstrated higher scores than men. A significant age class difference was observed only for depressive symptoms: the youngest age group (18–40 years) revealed higher values than the oldest group (> 50 years, p = .003). As compared to the general population, the physicians reported significantly elevated PHQ-2 (T1: M = 1.35, SD = 1.33; T2: M = 1.53, SD = 1.37; T3: M = 1.55, SD = 1.40; T4: M = 1.64, SD = 1.34) and GAD-2 scores (T1: M = 1.35, SD = 1.42; T2: M = 1.59, SD = 1.43; T3: M = 1.61, SD = 1.57; T4: M = 1.49, SD = 1.46) for all measurement points (all p < .001). The frequencies of probable depression (PHQ-2 ≥ 3) and anxiety (GAD-2 ≥ 3) were: 14.1% and 17.0% (T1), 16.5% and 21.9% (T2), 17.8% and 22.6% (T3) and 18.5% and 17.3% (T4), respectively.

**Conclusions:**

Mental distress of physicians in German hospitals has increased in the course of the COVID-19 pandemic with gender and age-related differences. Possible causes should be explored and regular monitoring of mental health and prevention programmes for physicians should be established.

**Trial registration:**

The study was registered on ClinicalTrials (DRKS-ID: DRKS00021268) on 9.4.2020.

**Supplementary Information:**

The online version contains supplementary material available at 10.1186/s40359-023-01354-5.

## Introduction

The prolonged stress experience during the COVID-19 pandemic had a negative impact on the mental health and well-being of the general population worldwide and especially of vulnerable groups such as individuals with preexisting mental or physical disorders, younger individuals and women [1, 2]. In the pandemic, health care workers (HCW) are confronted with additional long-term distress like higher risk of becoming infected or infecting their family, moral distress and continuous high workload. Global evidence shows high prevalence rates of common mental disorders among HCW such as depression, anxiety and posttraumatic stress disorder [3, 4]. A substantial proportion of physicians demonstrate elevated mental burden [5], while the prevalences for depression in the general population are lower [6].

However, the mental health of physicians and other HCW is not only in times of a pandemic at risk. Several systematic reviews and meta-analyses demonstrated high rates of depression in health care professionals before the COVID-19 pandemic, e.g. a pooled prevalence of depression or depressive symptoms of 28.8% was reported for resident physicians [7] and of 27.2% for medical students [8]. Besides, high levels of emotional exhaustion among psychiatrists [9] and other mental health professionals have been demonstrated [10].

A large body of cross-sectional studies exist in the meanwhile that have investigated several indicators of psychological distress among HCW during the COVID-19 pandemic, in particular of physicians and nurses. In comparison, longitudinal studies that analyze the long-term effects of the pandemic are scarce. Most of these were conducted in the USA [11–22].

Longitudinal studies on anxiety, depression and burnout among physicians consistently reveal an increase of the symptoms and prevalence rates as compared with pre-pandemic values (burnout: [20, 23]; anxiety: [12]; depression: [14]). When focusing differences between the course of depressive and anxiety symptoms during the pandemic, research shows conflicting results. In two large samples of HCW including physicians in Spain [24] and in Italy [25] a significant decrease of depressive and anxiety symptoms was detected, while another investigation in Italy with anaesthetists [26] reported a significant increase of depressive symptoms and no change concerning anxiety symptoms. Prospective studies in the USA most frequently demonstrated a reduction of anxiety symptoms or prevalence rates [11, 15, 17–19] and no change for depressive symptoms [15, 18, 22]. In a German study an increase of the prevalence rates for depression and a decrease for anxiety among physicians was observed [27].

To sum up, while the findings are not overall consistent, the majority of the studies demonstrated a significant decrease of initially high anxiety symptoms, but not of depression in the course of the pandemic. Almost in all studies, female gender [11, 13, 14, 21, 24] and younger age [13, 24, 28, 29] were associated with higher anxiety and depression symptoms. Most studies had two measurement points with a short period of time (mostly < one year) and have analyzed mixed samples of HCW. Nurses frequently revealed an increased psychological distress in comparison with physicians [20, 28] and HCW as compared with non-HCW [30].

To the best of our knowledge, our study is the first that has investigated the development of depressive and anxiety symptoms among physicians over the course of two years during the COVID-19 pandemic. The aims of the present study were to examine:

1. the course of levels of depressive and anxiety symptoms among physicians in hospitals in Germany during the COVID-19 pandemic between 2020 and 2022;
2. differences in these trajectories regarding gender, age and department type;
3. the levels of depressive and anxiety symptoms at all four measurement points in comparison with the German general population during the COVID-19 pandemic;
4. the frequency of clinically significant levels of depressive and anxiety symptoms (cut-off ≥ 3).

## Method

### Statement of ethics

The present study was conducted according to Declaration of Helsinki principles and was approved by the Ethics Committee of the Medical Faculty of the Friedrich-Alexander University Erlangen-Nürnberg (FAU) and other relevant participating university hospitals (Ethics Committee of the Medical Faculty of the Rheinische Friedrich Wilhelm University Bonn and Ethics Committee of the University Ulm). The study was registered on ClinicalTrials (DRKS-ID: DRKS00021268). All respondents provided their online informed consent for each survey.

### Data collection

The web-based survey was conducted by the psychosomatic departments of the university hospitals of Erlangen, Bonn, Ulm, Cologne, and Dresden at four measurement points (T1: April to July 2020, T2: November 2020 to January 2021, T3: May to July 2021, T4: February to May 2022). Further hospitals and various professional organizations and online platforms also promoted participation in the study. The survey was shared via mailing lists with all hospital staff, and via professional online platforms. It was programmed with two academic online survey tools, Unipark (www.unipark.com) and SoSci Survey (www.soscisurvey.de). It took approximately 15 min to complete the survey. The large part of the survey remained the same at all measurement points; but some items were added or removed due to special pandemic-related issues.

At each measurement point it was possible to newly participate in our study because it was also our intention to recruit large cross-sectional samples. Persons participating at several time points could be identified by a code that was unique for each participant and identical at different time points. At the beginning of the survey, each participant was asked to create a code consisting of the day of birth, the first letter of the place of birth, the first letter of his or her own first name, the first and last letter of the mother’s first name, and the first and last letter of the father’s first name.

Inclusion criteria were a minimum age of 18 years, working in the health care sector, residence/working place in Germany, and sufficient German language skills. Additional inclusion criteria for the analyses presented in this paper were the participation on at least two measurement points, belonging to the profession group of physicians and working in hospitals.

### Measures

#### Sociodemographic, occupational, and COVID-19-related variables

The following data were assessed: gender, age class, living alone, having children and migration background, department type, years of professional experience, and employment status, infection with SARS-CoV-2 virus, having direct contact at work with COVID-19 infected patients (proven by test), and transfer to another department due to the pandemic.

#### Depressive and anxiety symptoms

Depressive symptoms were assessed with the Patient Health Questionnaire-2 (PHQ-2) and general anxiety symptoms with the Generalized Anxiety Disorder Scale-2 (GAD-2) [31]. Sum scores range from 0 to 6, respectively. A cut-off-value of ≥ 3 has been suggested to detect probable cases of clinically significant levels of depressive and anxiety symptoms. In the present study, the validated German version obtained following Cronbach´s Alpha scores for the PHQ-2: T1 = 0.72; T2 = 0.73; T3 = 0.78; T4 = 0.72 and for the GAD-2: T1 = 0.76; T2 = 0.78; T3 = 0.80 and T4 = 0.73.

The online survey also included questionnaires measuring other constructs such as e.g. psychosocial resources. The corresponding results will be analyzed in other publications.

### Statistical analysis

Data analyses were performed with the programming language R V 4.2.0 (R Core Team, 2022) and SPSS V. 28 (IBM Corporation, Armonk, New York). To describe the sociodemographic, occupation-related and pandemic-related characteristics of the total sample, descriptive statistics (absolute and relative frequencies) were computed. Linear mixed regression models were calculated to analyze the association between the independent variables time point and gender or age classes, respectively, with the dependent variables depressive and generalized anxiety symptoms, respectively. Based on visual inspection, we also modelled the interaction between gender and time point, where appropriate. A cut-off-value of ≥ 3 in the PHQ-2 and in the GAD-2 was used to detect probable cases of depression and anxiety. Comparisons between observed values and published norm values in the general population were done for both, PHQ-2 and GAD-2 at all four time points using one-sample t tests. The corresponding effect sizes (Cohen´s d) were also reported (d ≥ 0.2 = small, d ≥ 0.5 = medium and d ≥ 0.8 = large effect size). The level of significance was set to p < .05 (two-sided).

## Results

A total of N = 2521 persons working in the health care sector participated at least at two measurement points of the web-based survey. In this paper, we focus on data from physicians working in the hospital setting (university hospitals and further hospitals of tertiary care). In total, N = 2287 physicians participated only once (T1: 794; T2: 800; T3: 297; T4: 396) (with completed PHQ-4). N = 340 physicians working in hospitals took part in the study at least at two measurement points with completed PHQ-4. Three assessments were achieved by n = 75 and all four by n = 22 physicians. In Supplement 1 the different combinations for the participation pattern of the physicians are presented for the study sample (N = 340).

### Sociodemographic, occupational and COVID-19-related variables

The sociodemographic, occupation- and COVID-19-related variables of the study sample for all measurement points are presented in Table 1. At each assessment, women presented approximately two thirds and young physicians (< 41 years) approximately half of the corresponding sample. The most noticeable difference between the four measurement points was observed concerning the proportion of physicians having been infected with the SARS-CoV-2 virus: 2–4% between T1 and T3 and 23% at T4.

**Table 1:** Socio-demographic, occupational and COVID-19-related characteristics of the study sample at four measurement points

	T1 (April to July 2020)	T2 (November 2020 to January 2021)	T3 (May to July 2021)	T4 (February to May 2022)
	**n = 206**	**n = 279**	**n = 146**	**n = 168**
**Gender, n (%)**				
Women	140 (68.0)	179 (64.2)	102 (69.9)	107 (63.7)
Men	66 (32.0)	100 (35.8)	44 (30.1)	61 (36.3)
**Age, years, n (%)**				
18-40	101 (49.0)	144 (51.6)	72 (49.3)	72 (42.9)
41-50	54 (26.2)	68 (24.4)	40 (27.4)	48 (28.6)
>50	51 (24.8)	67 (24.0)	34 (23.3)	48 (28.6)
**Living alone, n (%)**				
Yes	40 (19.4)	46 (16.5)	22 (15.1)	24 (14.3)
No	166 (80.6)	233 (83.5)	124 (84.9)	144 (85.7)
**Children, n (%)**				
Yes	112 (54.4)	147 (52.7)	85 (58.2)	103 (61.3)
No	94 (45.6)	132 (47.3)	61 (41.8)	65 (38.7)
**Migration background, n (%)**				
Yes	27 (13.1)	34 (12.2)	18 (12.3)	17 (10.1)
No	179 (86.9)	245 (87.8)	128 (87.7)	151 (89.9)
**Department type, n (%)**				
Operative	29 (14.1)	37 (13.3)	13 (8.9)	12 (7.1)
Conservative	55 (26.7)	82 (29.4)	49 (33.6)	47 (28.0)
Mixed operative conservative	24 (11.7)	28 (10.0)	16 (11.0)	20 (11.9)
Mental health	42 (20.4)	50 (17.9)	24 (16.4)	25 (14.9)
Intensive/emergency medicine	21 (10.2)	26 (9.3)	16 (11.0)	19 (11.3)
Anaesthesia	11 (5.3)	26 (9.3)	12 (8.2)	14 (8.3)
Occupational medicine	3 (1.5)	3 (1.1)		1 (0.6)
Other	21 (10.2)	27 (9.7)	16 (11.0)	16 (9.5)
Missing				14 (8.3)
**Professional Experience in Patient Care, n (%)**				
<3 years	28 (13.6)	36 (12.9)	13 (8.9)	9 (5.4)
3-6 years	25 (12.1)	41 (14.7)	22 (15.1)	23 (13.7)
>6 years	146 (70.9)	194 (69.5)	103 (70.5)	125 (74.4)
Not in patient care	7 (3.4)	8 (2.9)	8 (5.5)	4 (2.4)
Missing				7 (4.2)
**Employment**				
Full-time	143 (69.4)	200 (71.7)	99 (67.8)	110 (65.5)
Part-time	63 (30.6)	79 (28.3)	47 (32.2)	58 (34.5)
**Infection with SARS-CoV-2 virus, n (%)**				
Yes	4 (1.9)	8 (2.9)	6 (4.1)	39 (23.2)
No/ I don’t know	202 (98.1)	271 (97.1)	140 (95.9)	129 (76.8)
**Contact with infected patients, n (%)**				
Yes	98 (47.6)	155 (55.6)	43 (29.5)	110 (65.5)
No	108 (52.4)	124 (44.4)	103 (70.5)	58 (34.5)
**Transfer to another department due to the pandemic, n (%)**				
Yes	30 (14.6)	46 (16.5)	6 (4.1)	23 (13.7)
No	176 (85.4)	233 (83.5)	140 (95.9)	145 (86.3)

### Representativeness of the sample

In comparison with physicians working in the hospital setting registered in the statistics of the Federal Medical Association in Germany for 2020 [32] and 2021 [33], the women in our sample were overrepresented at all measurement points (63.7–69.9% vs. 48.4–48.5%). Concerning the age groups, our sample can be regarded as representative for physicians working in the hospital setting in Germany (18–40 years: 42.9–51.6% vs. 51.2–51.3%; 41–50 years: 24.4–28.6% vs. 22.1%; >50 years: 23.3–28.6% vs. 26.7%). Besides, also the proportion of physicians in our sample working in part-time (28.3–34.3%) is representative for Germany (28.9%) [34].

### Frequency of probable depression and anxiety

**Fig. 1:**
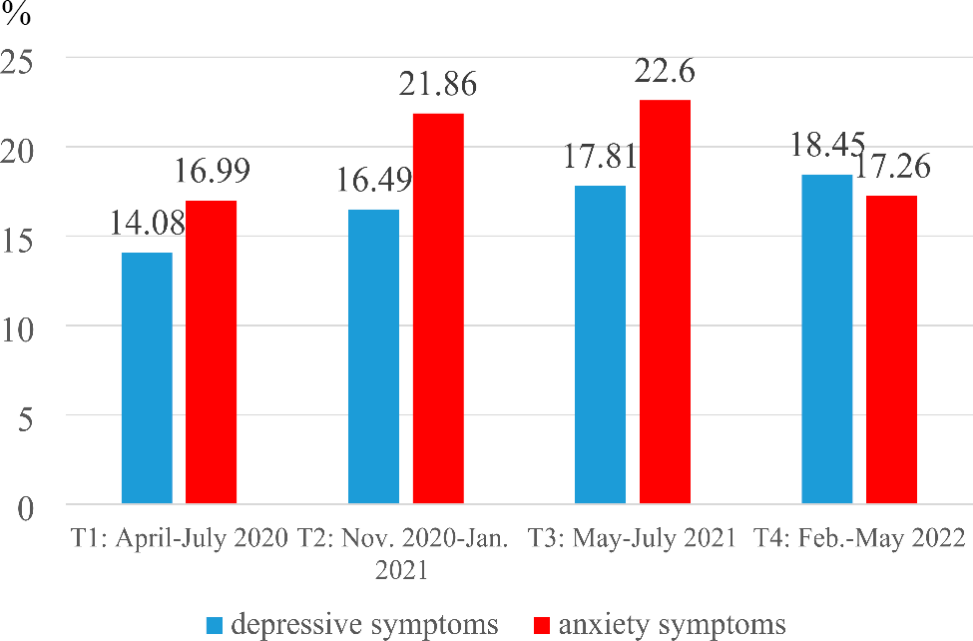
Frequency of probable depression and anxiety for T1 – T4 in the total study sample

The frequency of probable depression (cut-off-value of ≥ 3) was 14.08% (n = 29, T1), 16.49% (n = 46, T2), 17.81% (n = 26, T3) and 18.45% (n = 31, T4) (Fig. 1). The rate of probable anxiety (cut-off-value of ≥ 3) was 16.99% (n = 35, T1), 21.86% (n = 61, T2), 22.60% (n = 33, T3) and 17.26% (n = 29, T4) (Fig. 1).

### Course of depressive and anxiety symptoms during the pandemic in the total sample and in consideration of gender differences

**Fig. 2:**
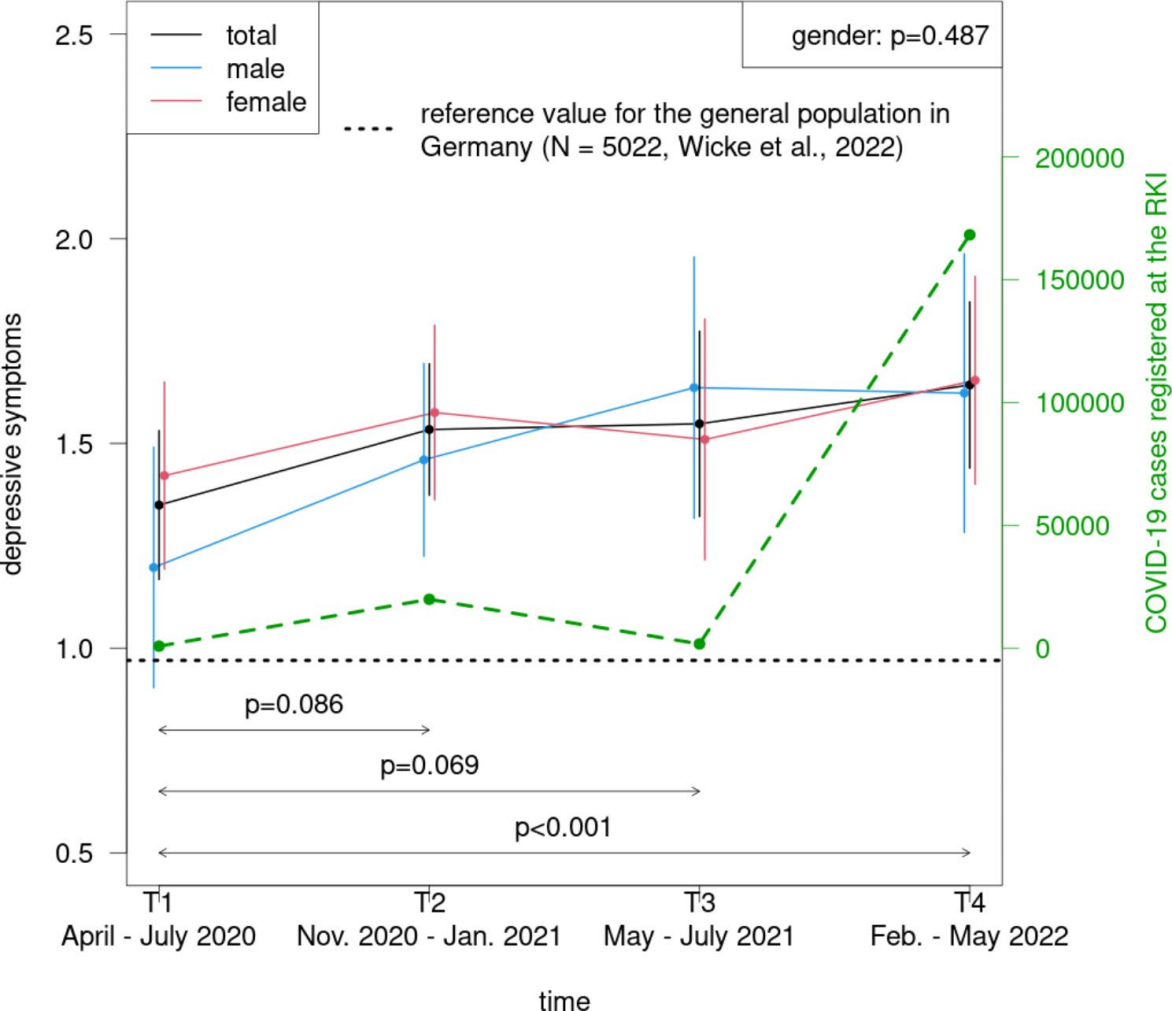
Course of depressive symptoms during the COVID-19 pandemic among physicians in dependence of gender

**Fig. 3:**
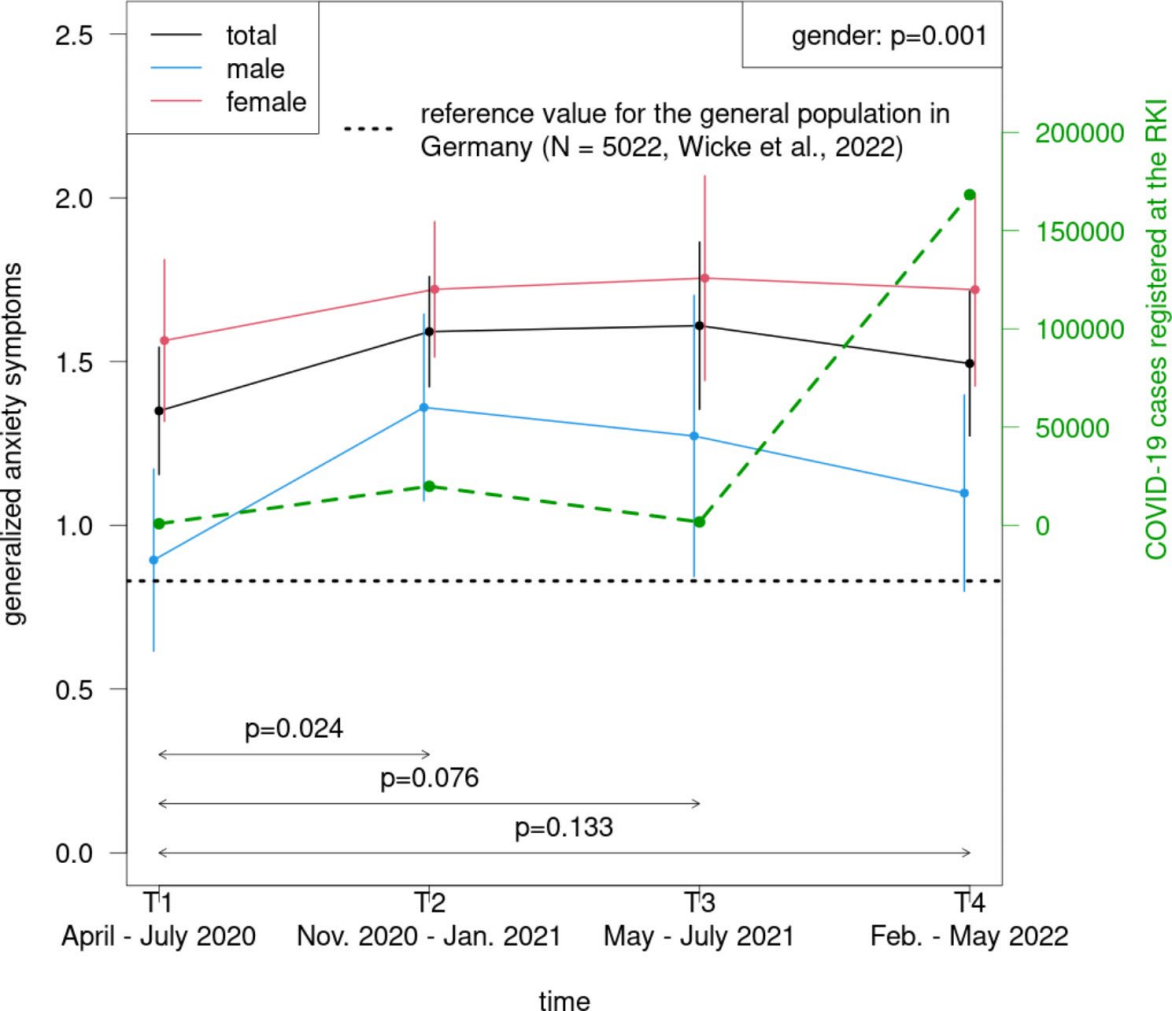
Course of generalized anxiety symptoms during the COVID-19 pandemic among physicians in dependence of gender

The physicians showed a significant increase of depressive symptoms from T1 to T4 (p < .001) and of anxiety symptoms from T1 to T2 (p = .024) (Fig. 2, Supplement 2 and 3). In comparison with T1, the depressive symptoms were elevated also at T2 and T3, however not significantly (T2: p = .086; T3: p = .069). A non-significant increase of anxiety levels was also observed at T3 (p = .076) (Fig. 3). The main effect of gender was not significant for depressive (p = .487), but for anxiety symptoms (p = .001): women demonstrated higher scores than men.

### Course of depressive and anxiety symptoms during the pandemic in consideration of age classes

**Fig. 4:**
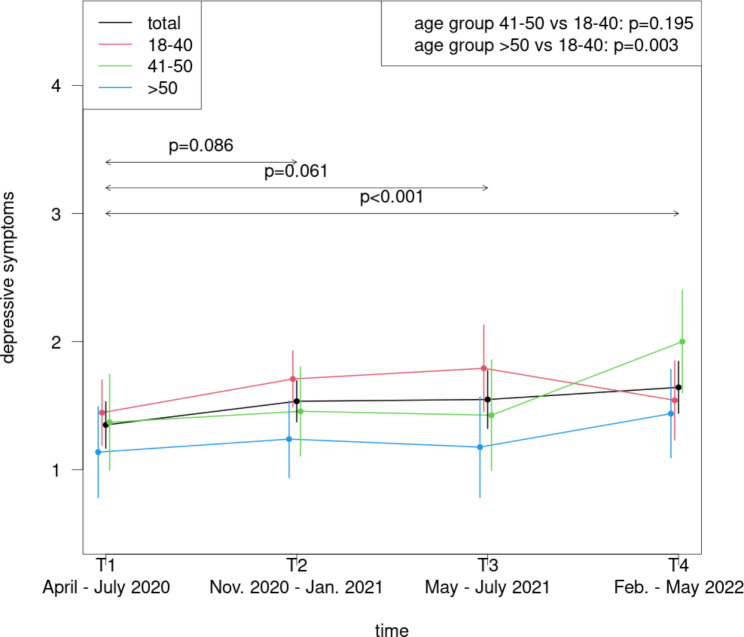
Course of depressive symptoms among physicians during the COVID-19 pandemic in dependence of age classes

**Fig. 5:**
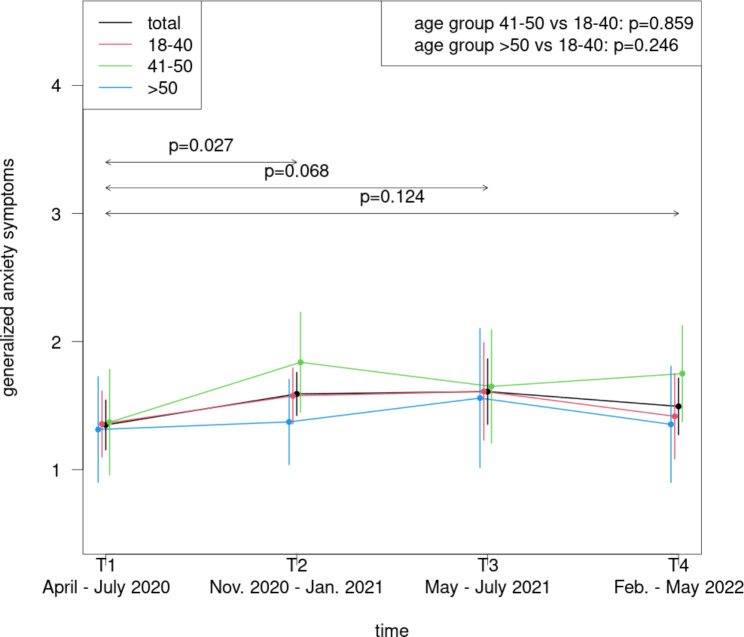
Course of anxiety symptoms during the COVID-19 pandemic among physicians in dependence of age classes

The youngest age group (18–40 years) revealed significantly increased depressive symptoms in relation to the oldest group (> 50 years) (p = .003) (Fig. 4); the difference between the youngest and the middle age group (41–50 years) was not significant (p = .195). No significant age class differences were detected for the anxiety symptoms (Fig. 5).

### Course of depressive and anxiety symptoms during the pandemic in dependence of department types

**Fig. 6:**
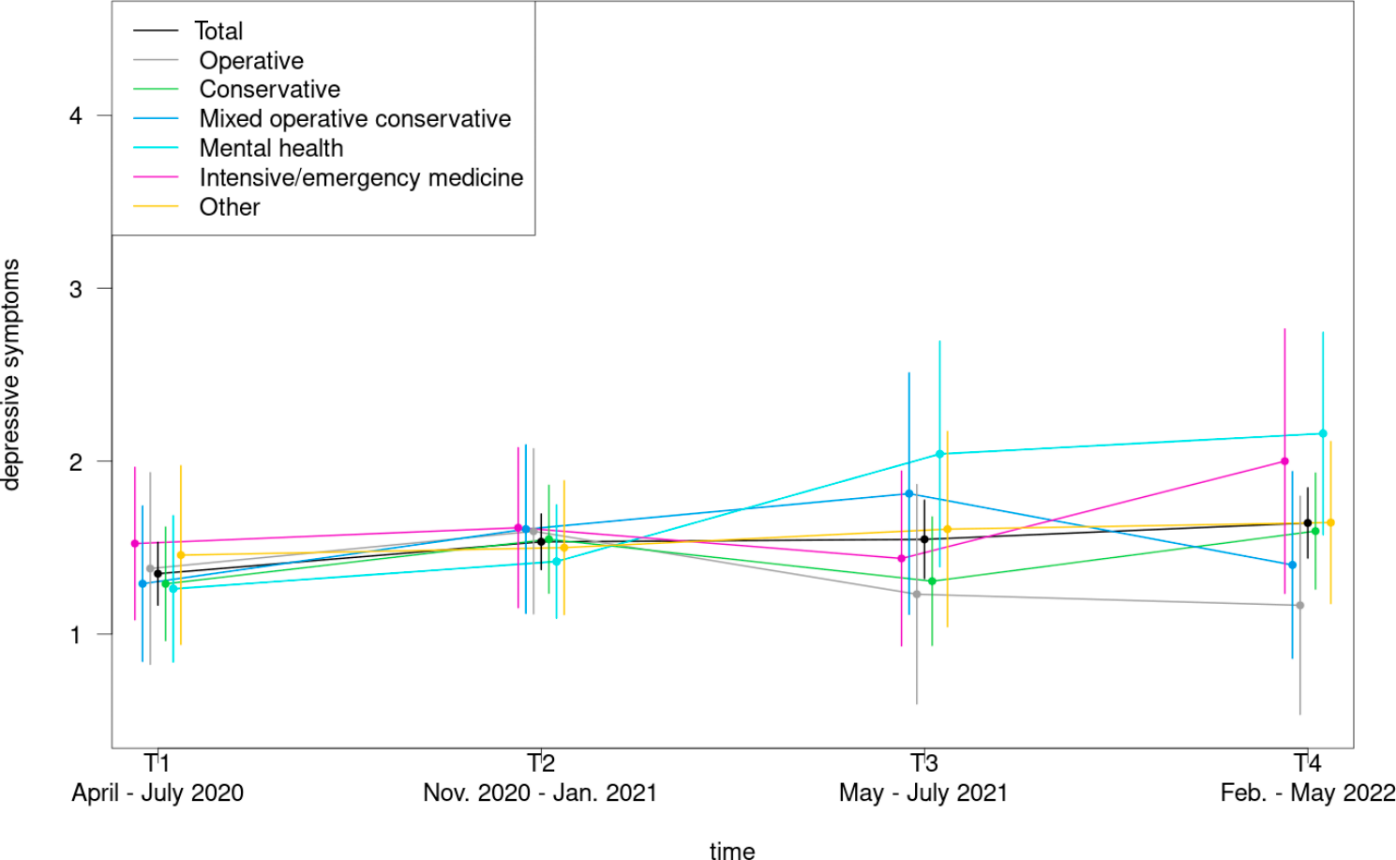
Course of depressive symptoms during the COVID-19 pandemic among physicians in dependence of department type

As presented in Fig. 6, from all examined medical departments physicians specialized in mental health revealed the lowest scores for depressive symptoms at T1 and T2, however the highest values at T3 and T4. Physicians working in the intensive/ emergency medicine demonstrated the highest levels of depressive symptoms at T1 and T2, while doctors from the operative setting showed the lowest symptomatology at T3 and T4.

**Fig. 7:**
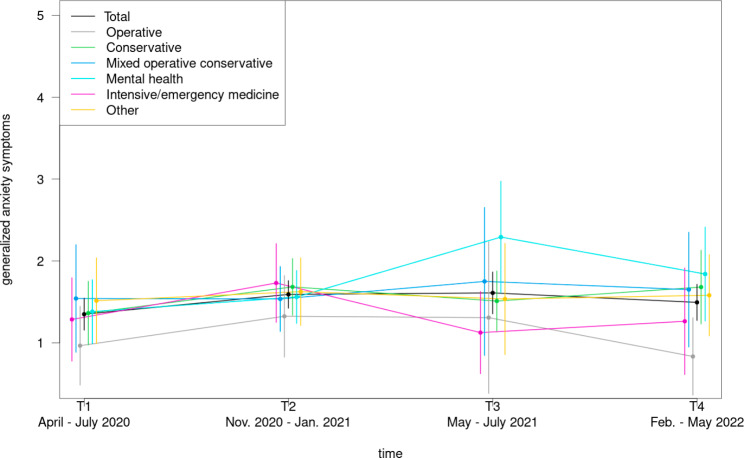
Course of anxiety symptoms during the COVID-19 pandemic among physicians in dependence of department type

Concerning the trajectory of anxiety symptoms physicians from mental health units also reported the highest scores for T3 and T4, while physicians from the intensive/emergency medicine as well as the operative departments revealed the lowest values for these measurement points (Fig. 7). The latter group also achieved the lowest anxiety levels at T1 and T2.

### Comparison with the German general population

As compared to values of the German general population during the pandemic as measured between 4 and 7/2020 and between 12/2020 and 2/2021 (depressive symptoms: M = 0.97 (SD = 1.21); anxiety symptoms: M = 0.83 (SD = 1.21) [35]), the physicians reported significantly elevated PHQ-2 and GAD-2 scores for all measurement points (all comparisons: p < .001, Cohen´s d between 0.29 and 0.53) (Figs. 2 and 3).

## Discussion

The present study investigated the trajectories of depressive and anxiety symptoms during the COVID-19 pandemic among physicians in hospitals. To the best of our knowledge, this is the first longitudinal assessment of the mental health of physicians over the course of two years during the pandemic. The first measurement point (April to July 2020) was conducted during the first wave of the COVID-19 pandemic in Germany, the second (November 2020 to January 2021) and the third (May to July 2021) each four months later and the fourth (February to May 2022) seven months in the aftermath of the third. The four assessments approximately correspond to the four pandemic waves. They took place in the time period after the peaks of each wave.

### The longitudinal change of depressive and anxiety symptoms

The main result of our study is the significant increase of depressive and anxiety symptoms among the physicians in the course of the COVID-19 pandemic. The deterioration of mental health is in line with some longitudinal studies (e.g. [28]. from Australia), while other surveys showed a significant improvement of symptoms among HCW (e.g. [24]. from Spain) or no change (e.g. [22]. from the USA, [36] from Switzerland). A possible explanation for the increase of symptoms may be a cumulative effect of different persistent physically and mentally challenging conditions during the pandemic at work and home, such as sustained high workload, time pressure, overtime hours, night-time work, fear of becoming infected or of infecting the family or colleagues and home schooling.

Not having enough time to rest may also negatively influence the mental health of physicians. In a previous cross-sectional analysis of the data at T1, we found insufficient recovery to be the strongest predictor for heightened depressive and one of the strongest predictors for enhanced anxiety symptoms [37].

Besides, the sustained increased depressive levels may also reflect a potential burnout. A significant association between depressive symptoms and burnout among physicians during the pandemic has been observed in a systematic review [38]. Longitudinal data suggest an increase of burnout rates among physicians and other HCW in the course of the COVID-19 pandemic [39] and also in relation with pre-pandemic assessments [40].

Analyzing the progress of the symptoms it is worth mentioning that the anxiety symptoms showed a more rapid increase than the depressive symptoms. This finding may be explained by the fact that in the early phase of the pandemic the knowledge about the pathophysiology of the virus was limited and the vaccines were not available resulting in elevated anxiety levels especially for those with contact to infected patients such as physicians. With the availability of the vaccines in the later course of the pandemic the anxiety levels may have lowered because immunized physicians felt able to better control the pandemic. However, the prolonged occupational and pandemic-related stress may have contributed to sustained elevated depressive symptoms.

### Gender-related differences

Another important result of our study concerned the significantly increased anxiety in women in relation to men, however, not regarding depressive symptoms. The elevated anxiety levels are consistent with results from other prospective studies among physicians [13, 14, 24]. In addition, they are also supported by a meta-analysis on gender and COVID-19 related fear and anxiety [41]. Independently of the pandemic, women are more vulnerable to most mental disorders than men [42].

The higher vulnerability for anxiety among women may be explained by biological (genetical and hormonal factors) and social factors (gender roles) or an interaction of both [41, 43]. The increased anxiety among women during the pandemic could also be (partially) attributed to a higher perception of COVID-19 risk [44] or of more responsibility perceived for the health of elderly relatives or children.

Nevertheless, the elevated vulnerability of women does not explain why there is no significant gender difference in depressive symptoms. The higher symptom severity in anxiety but not in depressive symptoms may be determined by cultural expectations towards gender roles making it more acceptable for women than for men to present anxiety or/and by biological factors.

### Age class-related differences

The youngest age group (18–40 years) presented significantly increased values in relation to the oldest group (> 50 years). The association between younger age and higher levels of depressive symptoms aligns with prior research with HCW [28, 29] as well as with general population [1, 2, 45]. Possible reasons for the prolonged better mental health of older physicians may be their greater resilience. Croghan and colleagues found higher resilience scores in older HCW of a general internal medicine division [46]. Due to more (occupational) experience the older professionals may have developed more effective strategies to cope with diverse stressors and thus maintain mental health.

Another explanation could also be the better economic status of older physicians in comparison with younger ones. There is empirical evidence that high economic status is a protective factor towards depression [47].

Finally, younger persons had greater access to social media. Being more connected with social media was proved to be a risk factor for higher rates of depressive and anxiety symptoms among physicians [48].

### Department type-related differences

Highest levels of depressive symptoms at T1 and T2 were detected in physicians in intensive/ emergency medicine units. These findings are in line with results observed in previous studies from the early phase of the COVID-19 pandemic showing medical professionals from high-risk departments and those directly engaged with care for patients with COVID-19 to be more likely to report mental health disorders such as depression and anxiety than HCW without direct health care of COVID-19 patients [49]. The highest depression levels in intensive/ emergency medicine clinics can be explained by the working conditions prevailing there like constant contact to highly infectious patients resulting in increased feelings of threat and also permanently high workload leading to exhaustion. Physicians working in the mental health setting demonstrated the lowest depressive scores at T1 and T2, but the highest at T3 and T4. The lowest levels of depressive symptoms in mental health physicians at T1 and T2 could be explained by their substantially decreased workload due to pandemic-related restrictions concerning the direct access of patients to clinics. For the highest severity of depressive symptoms at T3 and T4 the enlarged workload because of the increase of mental health problems as a consequence of the pandemic-associated stressors and therefore a dramatic escalation of the number of patients may be responsible. Another possible reason for the decreased levels of depressive symptoms in physicians from mental health units in the two first assessments may be better coping strategies with adversities due to the profession-specific expertise than among other medical specialties. As the differences between the different types of departments are presented only descriptively due to the small size of the sub-groups, future research should examine the unit-type-related differences regarding the trajectories of mental health symptoms in larger samples using appropriate significance tests.

### Comparison with the German general population

In comparison with the German general population during the COVID-19 pandemic, the physicians demonstrated significantly elevated PHQ-2 and GAD-2 scores for all measurement points. This finding is consistent with results from systematic reviews demonstrating higher prevalences for depression, anxiety, distress and other indicators of mental health among HCW in relation to the general population [50]. The prolonged worse mental health of the physicians may reflect the chronic stress of this profession in the course of the pandemic resulting from an accumulation of various stressors that may cause an exhaustion. Future research should investigate which factors are substantially responsible for the persisting increased mental burden of physicians during the pandemic as compared with the general population.

### Frequencies of probable depression and anxiety

The frequencies of probable depression for T1 and T2 in our study are comparable with data from a German longitudinal study including 87 physicians and nurses [27] with two measurement points similar to our two first assessments. The frequencies for a probable depression were 11.5% for T1 (April/May 2020) and 18.2% for T2 (November/December 2020), in our study the corresponding values were 14.1% and 16.5%. In comparison with the results of a recent systematic review and meta-analysis on the global prevalence of depression and anxiety among physicians based on studies from the first year of the COVID-19 pandemic (approximately two thirds of the included studies were from Asia and Europe) [5] our frequencies for T1 and T2 are slightly lower. A possible explanation could provide an analysis by Johns et al. [5] showing low prevalences of depression and anxiety for countries having > 30 physicians per 10.000 habitants (as in Germany, World Health Organization [51]). Systemic differences in the healthcare system between different countries may also explain the lower rates of probable depression and anxiety in our sample.

The frequencies for a probable depression detected in the present study are also lower than reported in a pre-pandemic meta-analysis on depression of resident physicians (28.8% [7]). The higher prevalence found in this meta-analysis could be attributed to methodological aspects (lower quality) of the included studies. The authors observed higher prevalence estimates in studies using less valid assessment methods and investigating less representative samples. Thus, it could be assumed that the pooled prevalence reported by Mata et al. [7] overestimates the actual pre-pandemic depression rate for physicians.

### Strengths and limitations

To the best of our knowledge, this is the first survey on mental health of physicians over a long period of time during the COVID-19 pandemic (two years) with four assessments in correspondence to the four pandemic waves (after the peak of each wave) and also presenting the course of mental health symptoms in dependence of gender, age classes and department types. Another strength of our study is the relatively large sample size: N = 340 physicians participated at least at two measurement points.

A limitation is the low response rate. Only a small number of physicians participated in all or in three measurement points. Thus, a selection bias cannot be excluded. Linear mixed regression (with random intercept in our case) can handle the problem that observations of subjects are not necessarily available at all four time points but nevertheless considers the fact that observations of the same person at different time points are not independent of each other. However, the fact that not all time points are available for all participants surely increases the uncertainty in our estimation. Possibly more physicians with a better mental health participated. This assumption is supported by findings from previous health surveys that mostly demonstrated a better health status among respondents than non-respondents (e.g. [52, 53]). Furthermore, the results of our study cannot be generalized to the group of all physicians of (university) hospitals. However, our sample can be regarded as representative for physicians in hospitals in Germany regarding some important socio-demographic and occupational characteristics. Finally, we did not recruit a control group from the general population. Future studies should explore the mental health of physicians using representative samples, clinical interviews and control groups from the general population to protect the health and thus to sustain the work ability of these key professionals during epidemics.

## Conclusion

It is evident from our longitudinal data that the levels of depressive and anxiety symptoms of physicians have increased in the course of the COVID-19 pandemic and were significantly elevated in comparison with the normal population in Germany during the pandemic at all measurement points. These findings underline the importance of a continuous monitoring of mental health of physicians during the pandemic and in its aftermath, e.g. in the form of a panel survey. The panel survey that examines the same individuals at several time points allows to collect a large amount of data with a broad spectrum of variables and to conduct causal analyses about the attitudes, behaviors, symptoms etc. of the target group; furthermore, panel surveys allows the investigation of intentions and their realization in later survey waves [54]. Thus, a panel survey of mental health of physicians could precisely analyze not only the course but also the reasons for increased distress and the role of specific protective factors of this profession. In addition, targeted interventions should be established to prevent a deterioration of the mental health of physicians. Female and younger physicians seem to be at higher risk for sustained elevated mental distress. Appropriate prevention programmes should be offered to these vulnerable groups to effectively cope with occupational and pandemic-related stressors. As proposed by Jacob et al. [55] these prevention programmes should aim at reducing social media use, strengthening social support, increasing healthy behaviors (e.g. physical activity) and decreasing unhealthy behaviors (e.g. alcohol consumption). Further studies should investigate the reasons, risk and protective factors for the sustained psychological symptoms among physicians.

### Electronic supplementary material

Below is the link to the electronic supplementary material.

Supplementary Material 1

Supplementary Material 2

Supplementary Material 3

## Data Availability

The data used for the current study are not publicly available due to ethical and legal data protection restrictions. The data presented in this study are available from the corresponding author on reasonable request.

## Abbreviations

GAD-2: Generalized Anxiety Disorder Scale-2
HCW: health care workers
M: mean
PHQ-2: Patient Health Questionnaire-2
SD: standard deviation
