# Supplementary figures and images for: Depressive and anxiety symptoms in the course of the COVID-19 pandemic among physicians in hospitals: results of the longitudinal, multicenter VOICE-EgePan survey over two years
Source: BMC Psychol. 2023 Oct 10;11:327. doi: 10.1186/s40359-023-01354-5 (PMC10566070; doi:10.1186/s40359-023-01354-5)

**Supplement 1:** Number of physicians participated in different measurement points.


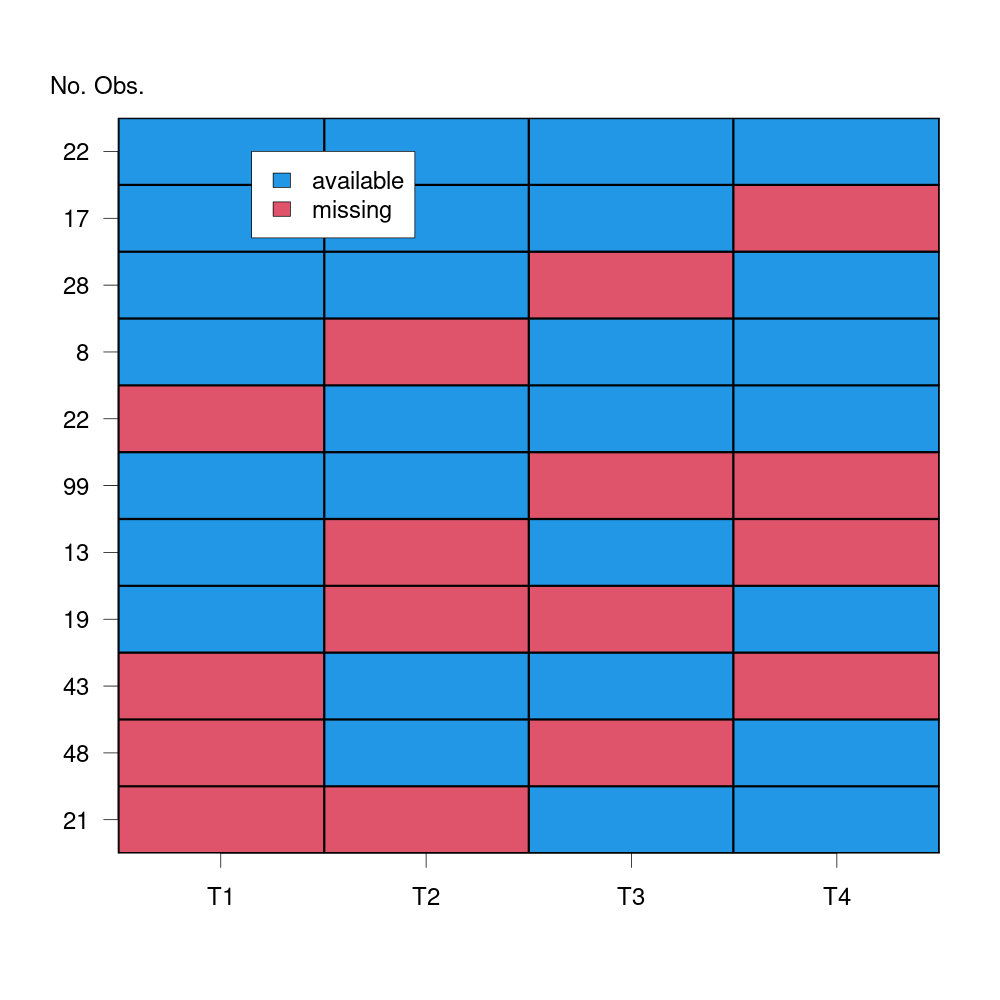

Supplement: Supplementary file 1 — Supplementary Material 1 [file 40359_2023_1354_MOESM1_ESM.docx]
