# Supplementary material for: Depressive and anxiety symptoms in the course of the COVID-19 pandemic among physicians in hospitals: results of the longitudinal, multicenter VOICE-EgePan survey over two years
Source: BMC Psychol. 2023 Oct 10;11:327. doi: 10.1186/s40359-023-01354-5 (PMC10566070; doi:10.1186/s40359-023-01354-5)
Supplement: Supplementary file 2 — Supplementary Material 2 [file 40359_2023_1354_MOESM2_ESM.docx]

**Supplemet 2:** Coefficients with corresponding confidence intervals and p-values for the four models presented in Figures 1-4.

**Coefficient 95% CI p-value**

**Depressive symptoms, gender**

(Intercept) 1.275 1.031; 1.519 <0.001

gender: f (ref.: m) 0.092 -0.167; 0.350 0.487

time: T2 (ref.: T1) 0.161 -0.022; 0.345 0.086

T3 0.214 -0.016; 0.444 0.069

T4 0.417 0.197; 0.635 <0.001

**Generalized anxiety symptoms, gender**

(Intercept) 1.038 0.778; 1.297 <0.001

gender: f (ref.: m) 0.500 0.220; 0.779 0.001

time: T2 (ref.: T1) 0.216 0.030; 0.402 0.024

T3 0.212 -0.021; 0.446 0.076

T4 0.170 -0.051; 0.392 0.133

**Depressive symptoms, age**

(Intercept) 1.487 1.278; 1.695 <0.001

age group: 41-50 (ref.: 18-40) -0.185 -0.466; 0.094 0.195

>50 -0.443 -0.734; -0.152 0.003

time: T2 (ref.: T1) 0.161 -0.022; 0.344 0.086

T3 0.220 -0.010; 0.449 0.061

T4 0.436 0.217; 0.654 <0.001

**Generalized anxiety symptoms, age**

(Intercept) 1.406 1.182; 1.630 <0.001

age group: 41-50 (ref.: 18-40) 0.028 -0.279; 0.333 0.859

>50 -0.190 -0.510; 0.130 0.246

time: T2 (ref.: T1) 0.211 0.025; 0.397 0.027

T3 0.219 -0.015; 0.453 0.068

T4 0.176 -0.047; 0.398 0.124
