# Supplementary material for: Depressive and anxiety symptoms in the course of the COVID-19 pandemic among physicians in hospitals: results of the longitudinal, multicenter VOICE-EgePan survey over two years
Source: BMC Psychol. 2023 Oct 10;11:327. doi: 10.1186/s40359-023-01354-5 (PMC10566070; doi:10.1186/s40359-023-01354-5)
Supplement: Supplementary file 3 — Supplementary Material 3 [file 40359_2023_1354_MOESM3_ESM.docx]

**Supplemet 3:** Mean values and standard deviations for the four models presented in Figures 1-4.

**Mean (SD) T1 Mean (SD) T2 Mean (SD) T3 Mean (SD) T4**

**Depressive symptoms**

Total 1.350 (1.330) 1.534 (1.367) 1.548 (1.395) 1.643 (1.342)

Male 1.197 (1.218) 1.460 (1.201) 1.636 (1.080) 1.623 (1.356)

Female 1.421 (1.378) 1.575 (1.453) 1.510 (1.514) 1.654 (1.340)

18-40 1.446 (1.307) 1.708 (1.348) 1.792 (1.463) 1.542 (1.342)

41-50 1.370 (1.405) 1.456 (1.470) 1.425 (1.394) 2.000 (1.414)

>50 1.137 (1.296) 1.239 (1.256) 1.176 (1.167) 1.438 (1.219)

**Generalized anxiety symptoms**

Total 1.350 (1.423) 1.591 (1.434) 1.610 (1.573) 1.494 (1.460)

Male 0.894 (1.152) 1.360 (1.453) 1.273 (1.453) 1.098 (1.193)

Female 1.564 (1.490) 1.721 (1.410) 1.755 (1.607) 1.720 (1.553)

18-40 1.356 (1.324) 1.576 (1.336) 1.611 (1.649) 1.417 (1.441)

41-50 1.370 (1.545) 1.838 (1.645) 1.650 (1.424) 1.750 (1.329)

>50 1.314 (1.503) 1.373 (1.391) 1.559 (1.618) 1.354 (1.604)
